# Oxygen Species Involved in Complete Oxidation of CH_4_ by SrFeO_3-δ_ in Chemical Looping Reforming of Methane

**DOI:** 10.3390/ma17133212

**Published:** 2024-07-01

**Authors:** Jianan Hao, Liuqing Yang, Junshe Zhang

**Affiliations:** School of Chemical Engineering and Technology, Xi’an Jiaotong University, Xi’an 710049, China; hjn3395@stu.xjtu.edu.cn (J.H.); 4120116024@stu.xjtu.edu.cn (L.Y.)

**Keywords:** methane reforming, redox cycle, perovskite, transient reduction, surface species

## Abstract

Compared with conventional methane reforming technologies, chemical looping reforming (CLR) has the advantages of self-elimination of coke, a suitable syngas ratio for certain down-stream processes, and a pure H_2_ or CO stream. In the reduction step of CLR, methane combustion has to be inhibited, which could be achieved by designing appropriate oxygen carriers and/or optimizing the operating conditions. To gain a further understanding of the combustion reaction, methane oxidation by perovskite (SrFeO_3-δ_) at 900 °C and 1 atm in a pulse mode was investigated in this work. The oxygen non-stoichiometry of SrFeO_3-δ_ prepared by a Pechini-type polymerizable complex method is 0.14 at ambient conditions, and it increases to 0.25 and subsequently to 0.5 when heating from 100 to 900 °C in argon that contains 2 ppmv of molecular oxygen. The activation energies of the first and second transitions are 294 and 177 kJ/mol, respectively. The presence of 0.99 vol.% hydrogen in argon significantly reduces the amount CO_2_ produced. At a pulse interval of 10 min, the amount of CO_2_ produced in the absence of hydrogen is one order of magnitude greater than that in the presence of hydrogen. In the former case, the amount of CO_2_ produced dramatically decreases first and then gradually approaches a constant, and the oxygen species involved in methane combustion can be partially replenished by extending the pulse interval, e.g., 82.5% of this type of oxygen species is replenished when the pulse interval is extended to 60 min. The restored species predominantly originate from those that reside in the surface layer or even in the bulk.

## 1. Introduction

At the end of 2023, global proven natural gas reserves have risen to an estimated 212.54 trillion cubic meters [1], which takes no account of those trapped in gas hydrates at the continental slopes of oceans and in permafrost areas; it was estimated that the amount of natural gas stored in global recoverable natural gas hydrates is between 25.55 and 84.36 trillion cubic meters [2]. Additionally, biogas is being considered a renewable natural gas that is generated from the thermochemical conversion or anaerobic digestion of crops, residues, and waste. Currently, a major portion of natural gas in the world is directly burned to produce energy for heating and power generation, which has high exergy losses and CO_2_ emission. 

Compared with combustion, the transformation of the predominant component of natural gas, methane (CH_4_), into chemicals or liquid fuels is more economically viable [3]. Methane can be converted to syngas, a mixture of hydrogen (H_2_), carbon monoxide (CO), and some carbon dioxide (CO_2_) by reforming [4]. Subsequently, syngas is used to produce high-value products, such as liquid fuels, waxes, or light alkenes. There are two main conventional reforming technologies: steam methane reforming (SMR) and dry reforming of methane (DRM) [5,6]. The former is being widely used in the chemical industry to produce hydrogen, whereas the latter has not yet reached full technological maturity, although it has environmental and economic advantages. CH_4_ has a symmetrical tetrahedral configuration with four equivalent C−H bonds, which makes it very difficult to activate methane. Thus, the reforming reactions are always carried out at high temperatures in the presence of supported metal catalysts. One of the thorny issues associated with thermal catalytic reforming at high temperatures and pressures is coking, which results from both methane thermolysis and CO disproportionation reactions. To address that, one can optimize the operating conditions and/or design novel catalysts [7,8]. Alternatively, coking is inherently circumvented in chemical looping reforming (CLR) processes [9,10,11], which can be driven by solar energy.

A typical chemical looping scheme involves two steps: a redox material (usually a metal oxide) is first reduced by methane (MeO_x_ + δCH_4_ → MeO_x-δ_ + 2δH_2_ + δCO), and then the reduced metal oxide is re-oxidized (or regenerated) by putting it in contact with steam or CO_2_ to replenish its lattice oxygen, producing hydrogen or carbon monoxide (MeO_x-δ_ + δH_2_O/CO_2_ → MeO_x_ + δH_2_/CO). Generally, coking may occur in the reduction step, but the solid carbon is completely gasified (C(s) + CO_2_ → 2CO, C(s) + H_2_O → H_2_ + CO) in the oxidation step. Another two obvious advantages of CLR over conventional reforming are: the syngas ratio (nH_2_:nCO ≈ 2:1) of the reduction step is very suitable for most downstream processes such as Fischer-Tropsch synthesis and direct methanol synthesis. A pure CO stream is obtained in the oxidation step if CO_2_ is used to replenish the lattice oxygen, which could be directly used as the feed for the production of phosgene as an intermediate for polycarbonate production [12]. Additionally, if steam is used to replenish the lattice oxygen, then a hydrogen-rich or even pure hydrogen stream will be produced in the oxidation step. The high concentration of hydrogen reduces the specific requirements of utilities and offers substantial advantages in the selection of purification technologies.

In the reduction step (with respect to the redox material), two main side reactions have to be inhibited: one is the complete oxidation of methane (combustion), and the other is methane pyrolysis. The former reduces carbon efficiency, whereas the latter results in coke. In typical operating conditions, gas-phase methane pyrolysis is always negligible, and catalytic pyrolysis over metal particles plays a key role in coking. Therefore, any strategies that can tune the geometric and electronic structure of the metal particles could suppress coking, e.g., designing the redox materials (also known as oxygen carriers) with confined microenvironments. On the one hand, considerable effort in recent years has been devoted to the development of high-performance perovskite-typed OCs. Li et al. reported perovskite nanocomposites where SrFeO_3-δ_ is dispersed into a matrix of mixed metal oxides and exhibit excellent redox performance toward the chemical looping and reforming of methane [13]. Shen et al. found that A and B co-doping of LaMnO_3-δ_ shows satisfactory performance and good stability in chemical looping steam methane reforming [14]. Our group investigated the effect of nickel and cobalt doping on the redox performance of SrFeO_3-δ_ toward chemical looping dry reforming of methane [15]. Zhou et al. prepared high-performance Sr_0.98_Fe_0.7_Co_0.3_O_3-δ_ perovskites by combining A-site defects and B-site doping of SrFeO_3-δ_ [16]. On the other hand, the mechanism of methane oxidation by perovskite-typed OCs remains elusive. Methane combustion is always attributed to adsorbed oxygen species on the surface of oxygen carriers (OCs) [14,17,18]. However, adsorbed oxygen species can enter the lattice structure, and oxygen species can leave the lattice structure after migrating to the surface. There is also evidence that surface species and lattice species can both be active in the same reaction, and the surface species operate on shorter time scales (<10 ms) [19]. Therefore, it is worthwhile to investigate the oxygen species that are involved in the deep oxidation of methane in detail. In this context, we investigated methane oxidation by SrFeO_3-δ_, a perovskite-typed OC, at 900 °C and 1 atm in a pulse mode. The OC was synthesized by a Pechini-type polymerizable complex method, and the carrier gas was pure argon or an argon and hydrogen mixture. At a pulse interval of 10 min, the amount of CO_2_ produced in the absence of hydrogen is one order of magnitude greater than that in the presence of hydrogen. In the former case, the amount of CO_2_ produced dramatically decreases first and then gradually approaches a constant, and the oxygen species involved in methane combustion can be partially replenished by extending the pulse interval. Our findings suggest that most oxygen species that perform methane combustion reside in the surface layer, and a large portion of this kind of oxygen species can be eliminated by hydrogen. These findings further our understanding of e methane combustion in the reduction step of chemical looping reforming.

## 2. Experimental

### 2.1. Materials

The chemicals used in preparing perovskite were Fe(NO_3_)_3_·9H_2_O (98.5%), Sr(NO_3_)_2_ (99.5%), citric acid (C_6_H_8_O_7_, CA, 99.5%), and ethylene glycol (C_2_H_6_O_2_, EG, 99.0%). All chemicals were purchased from Sinopharm. The deionized (DI) water was provided by Tianyi, Inc., Xi’an, China. The gases used for methane oxidation were argon (Ar, 99.999%), hydrogen (H_2_, 99.99%), and methane (CH4, 99.999%), which were supplied by Tenglong, Inc., Xi’an, China.

### 2.2. Synthesis of SrFeO_3-δ_

The oxygen carrier was synthesized by the Pechini-type polymerizable complex method. Specifically, stoichiometric amounts of Sr(NO_3_)_2_ and Fe(NO_3_)_3_∙9H_2_O as well as CA (the mole ratio of CA to metal cations was set to 2.5) were dissolved in DI water. After stirring the mixture for 30 min at 40 °C, EG (the mole ratio of EG to CA was adjusted to 1.5) was added to the resulting solution, followed by raising the temperature of the mixture to 80 °C. The mixture was kept at this temperature under agitation until a syrup was formed. Subsequently, the gel was dried at 110 °C in an oven for 10 h to remove the residual water. After that, the sample was heated to 400 °C for 2 h and then calcined at 1200 °C for 10 h in air flow. The residue was ground and sieved into 250–450 µm for testing.

### 2.3. Characterization

The XRD patterns of the as-prepared sample was collected on a D/Max-R diffractometer (XRD 6100 Lab, Shimadzu, Japan) equipped with a Cu Kα radiation source (λ = 0.15406 nm) with the operating voltage and current of 40 kV and 30 mA, respectively. The sample was scanned over a 2θ range of 10−90° with a scan rate of 5 °/min under ambient conditions.

### 2.4. Oxygen Release and Methane Oxidation

Oxygen release and methane oxidation were carried out on an in-house reaction unit at 1 atm. The flowrates of argon and hydrogen were controlled by mass flow controllers (ALICAT KM3100, Alicat Scientific, Tucson, AZ, USA). After loading 0.35 g of as-prepared OC particles into a quartz tubular reactor (ID = 8 mm, the particles were placed between two quartz wool plugs) that was vertically inserted into an electric furnace (MTI OTF1200X, Hefei Kejing Material Technology Co., Ltd., Hefei, China) with a K-type thermocouple monitoring the furnace temperature, the reactor was purged with 100 STP mL/min Ar. After the signal of argon stabilized, the reactor was heated up to 900 °C at ramping rates of 2.5, 5, or 10 °C/min. The reactor was kept at the targeted temperature for 1 h before injecting methane through a six-way valve with a loop volume of 1.37 mL; the loop temperature was close to 25 °C and the pressure of methane was 1 atm. The composition of the output stream was continuously monitored by a quadrupole mass spectrometer (Pfeiffer OmniStar GSD320, Pfeiffer Vacuum, Germany). The amounts of methane unreacted and hydrogen and CO_2_ produced in any pulses were calculated from the areas of m/z signals at 15, 2, and 44, respectively. When hydrogen was introduced into argon, its flowrate was set to 1.00 STP mL/min. Methane conversion and CO_2_ selectivity were calculated by the following equations,

(1) $$X_{CH_{4}}=\frac{n_{CH_{4},in}-n_{CH_{4},out}}{n_{CH_{4},in}}\times 100\%$$

(2) $$S_{CO_{2}}=\frac{n_{CO2,out}}{n_{CH_{4},in}-n_{CH_{4},out}}\times 100\%$$

where n_CH4,in_ is the amount of methane injected into the reactor (μmol), n_CH4,out_ is the amount of methane unreacted in a pulse (μmol), n_CO2,out_ is the amount of CO_2_ produced in a pulse (μmol).

## 3. Results and Discussion

**Figure 1:**
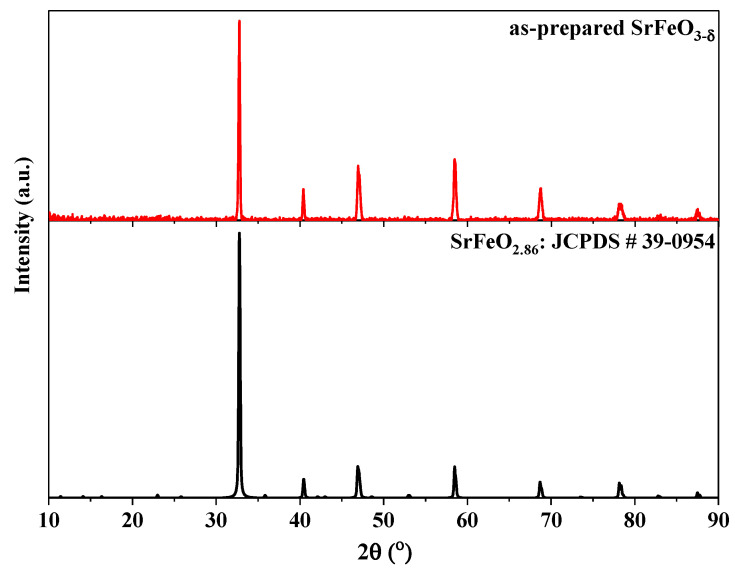
X-ray diffraction patterns of as-prepared SrFeO_3-δ_ at ambient conditions.

The compound SrFeO_3-δ_ belongs to the class of non-stoichiometric compounds in which the oxygen non-stoichiometry (δ) is variable [20,21,22]. This compound, δ can continuously vary in the range of 0 to 0.5. Extensive studies on this compound have shown that there exist four phases of ideal composition in the range 0 ≤ δ ≤ 0.5 [18,19], these are: cubic SrFeO_3_ (δ = 0), tetragonal SrFeO_2.875_ (δ = 0.125; or equivalently Sr_8_Fe_8_O_23_), orthorhombic SrFeO_2.75_ (δ = 0.25; or equivalently Sr_4_Fe_4_O_11_), and brownmillerite SrFeO_2.5_ (δ = 0.5; or equivalently Sr_2_Fe_2_O_5_). For all the others, the composition is a mixture of the two nearest ideal phases. On the other hand, the ranges of δ in which a single phase exists at room temperature differ from one study to another and can be roughly estimated to be 0 ≤ δ ≤ 0.08 for cubic, 0.125 ≤ δ ≤ 0.17 for tetragonal, and 0.25 ≤ δ ≤ 0.32 for orthorhombic phase. For the as-prepared SrFeO_3-δ_ at ambient conditions, its diffraction patterns well match those of SrFeO_2.86_ (Figure 1), suggesting that δ is equal to 0.14. From the above description, it can be said that the as-prepared SrFeO_3-δ_ has a tetragonal structure and it may contain a minor amount of orthorhombic phase.

**Figure 2:**
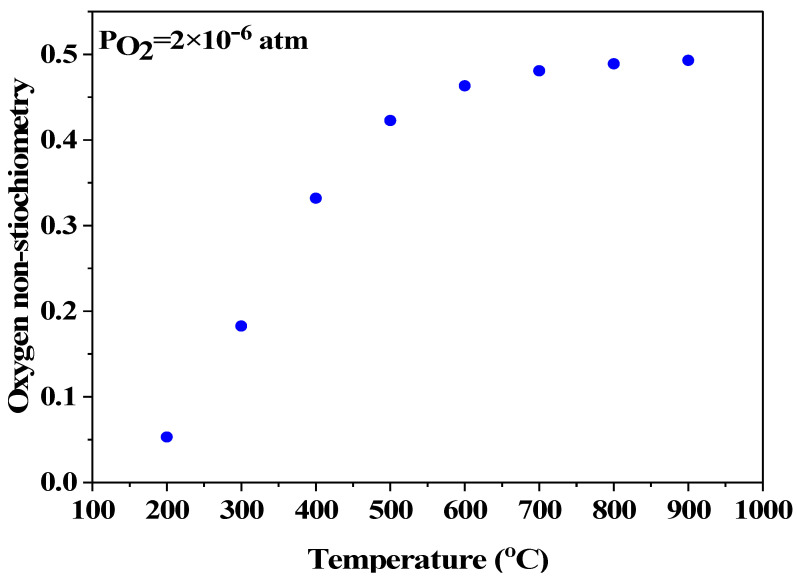
Oxygen non-stoichiometry of SrFeO_3-δ_ as function of temperature.

For SrFeO_3-δ_, the oxygen non-stoichiometry depends on both temperature and oxygen pressure and the relationship can be described by the following equations [20,21,22],

(3) $$-RTlnK_{O}=∆H_{O}^{o}-T∆S_{O}^{o}=-100000+88T$$

(4) $$K_{O}=\frac{\left(3-\delta \right)\times {\left(1-2\delta \right)}^{2}}{P_{O_{2}}^{1/2}\times 4\delta^{3}}$$

where T is temperature (K), P_O2_ is oxygen pressure (atm). According to the specification provided by the vendor, the oxygen content in the pure argon is less than 1 ppmv but the measured one is 2 ppmv. In this case, the variation of the oxygen non-stoichiometry with temperature is illustrated in Figure 2. As can be seen, δ approaches 0.5 as the temperature increases to 900 °C, suggesting that the tetragonal structure will change to brownmillerite phase when SrFeO_3-δ_ is treated in pure argon at 900 °C.

**Figure 3:**
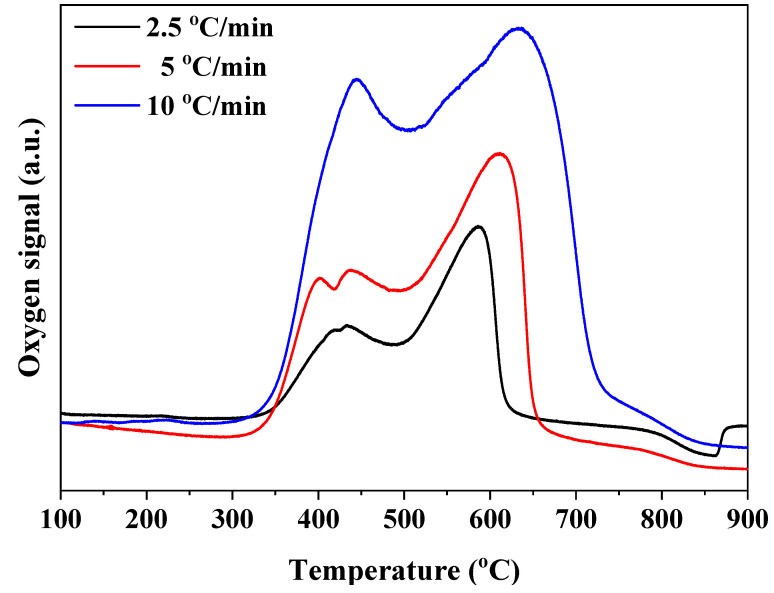
Oxygen release from SrFeO_3-δ_ in argon at three ramping rates: total pressure is 1 atm and the partial pressure of molecular oxygen is about 2 × 10^−6^ atm.

Before pulsing methane to the OC bed, as-prepared SrFeO_3-δ_ particles were heated in pure argon to the targeted temperature. As Figure 3 shows, oxygen release was observed at a temperature range of 300 to 800 °C. The release of oxygen can be divided into two steps, one at temperatures between 300 and 500 °C and the other at temperatures between 500 and 800 °C. At ramping rates of 2.5 and 5 °C/min, there exist doublet peaks in the low-temperature region that merge into a single peak when the ramping rate was 10 °C, but no trend can be observed. Therefore, this might come from the fluctuation in the flowrate of argon. Another observation is that the amount of oxygen released in the low-temperature region is less than that in the high-temperature one. Specifically, the area of the high-temperature peak is 2.5 times as much as that of the low-temperature one at a ramping rate of 2.5 °C/min. 

**Figure 4:**
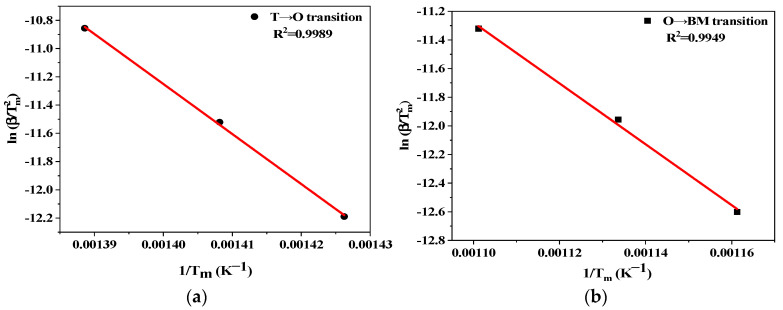
(**a**) Kissinger plots for tetragonal SrFeO_2.86_ to orthorhombic SrFeO_2.75_ transition; (**b**) orthorhombic SrFeO_2.75_ to brownmillerite SrFeO_2.5_ transition in argon.

Based on the above description, SrFeO_2.86_ first transforms into SrFeO_2.75_ at low temperatures and then into SrFeO_2.5_ at high temperatures as the perovskite is heated up from 100 to 900 °C. As a result, the amount of oxygen released in the high-temperature region is 2.3 times as much as that in the low-temperature region. Upon combination of this value and the measured one, we can conclude that the as-prepared sample undergoes two consecutive phase transitions in the pretreatment, i.e., tetragonal SrFeO_2.86_ to orthorhombic SrFeO_2.75_ (T→O transition) and then to brownmillerite SrFeO_2.5_ (O→BM transition). Furthermore, the activation energy of these two transformations can be evaluated using the Kissinger method [23,24], which is generally performed considering that

(5) $$\ln\left(\frac{\beta }{T_{m}^{2}}\right)=\ln\left(\frac{AR}{1000E}\right)-\left(\frac{1000E}{R}\right)\frac{1}{T_{m}}$$

where *β* is the ramping rate (°C/min), *A* is the pre-exponential factor, *R* is the gas constant; *E* is the activation energy (kJ/mol), *T*_m_ is the temperature at which the reaction rate is maximum (K). The plots of ln[*β*/*T*_m_^2^] vs. 1/*T*_m_ are presented in Figure 4, from which the values of activation energy for T→O and O→BM transitions are obtained from the slope of straight lines; they are 294 and 177 kJ/mol, respectively. 

Previous studies have shown that SrFeO_3-δ_ can be reduced to SrO and metallic iron, which are subsequently oxidized back to SrFeO_3-δ_ at high temperatures in a redox mode [13,18]. As previously stated, brownmillerite SrFeO_2.5_ is the stable phase at 900 °C in pure argon. Thus, the following equation can be written for the redox reaction,

(6) $$\frac{4}{3}SrO+\frac{4}{3}Fe+O_{2}=\frac{4}{3}SrFeO_{2.5}$$

**Figure 5:**
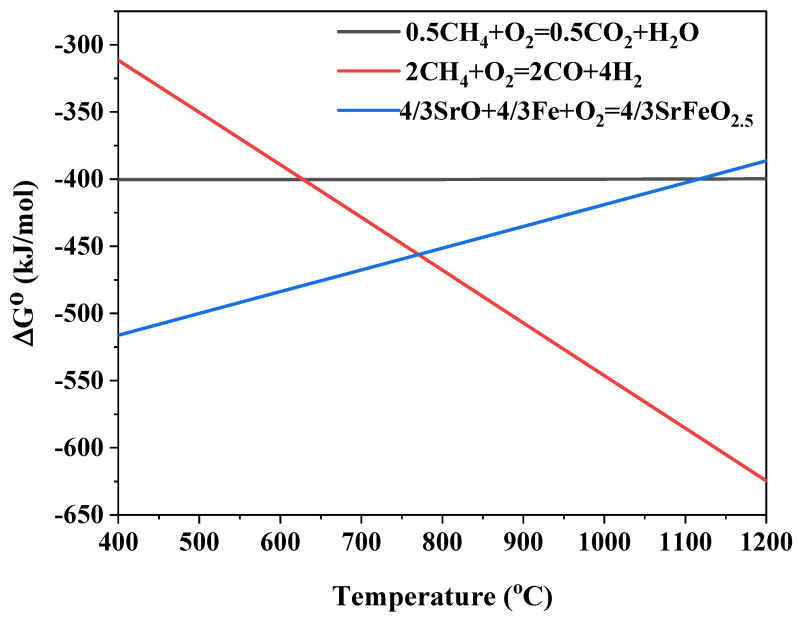
The standard Gibbs-energy change for methane oxidation and SeFeO_2.5_ redox reactions as a function of temperature.

The standard Gibbs-energy changes of the above reaction and methane oxidation as a function of temperature are given in Figure 5. According to the standard Gibbs-energy change in reactions, the reduction of SrFeO_2.5_ by methane can be divided into three regions: neither methane combustion nor partial oxidation of methane is thermodynamically favorable at temperatures below 770 °C, only partial oxidation of methane is thermodynamically favorable at temperatures between 770 and 1110 °C, and both are thermodynamically favorable at temperatures above 1110 °C. Therefore, chemical looping reforming of methane should be operated in the second region. At 900 °C and 1 atm, the partial pressure of oxygen is 4.2 × 10^−20^ atm at equilibrium; thus, the equilibrium ratio of *P*_CO_ to *P*_CO2_ is about 37.4. It is worth noting that the lower the oxygen pressure, the higher the selectivity toward partial oxidation, and the lower the conversion in the second region.

**Figure 6:**
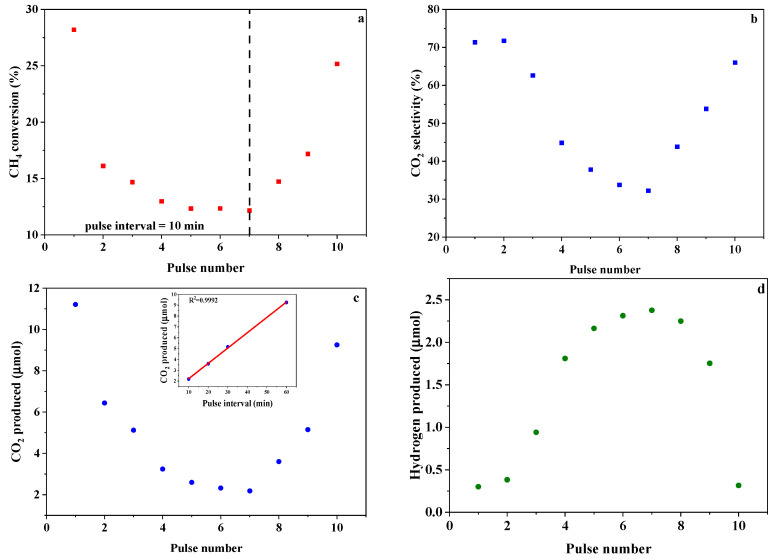
Methane oxidation by SrFeO_2.5_ at 900 °C and 1 atm in a pulse mode: m_OC_ = 0.35 g, F_Ar_ = 100 STP mL/min, n_CH4,injected_ = 55.7 μmol, the interval of 8, 9, and 10 pulses = 20, 30, 60 min, respectively. (**a**) CH_4_ conversion (%), (**b**) CO_2_ selectivity (%), (**c**) CO_2_ produced (μmol), (**d**) Hydrogen produced (μmol).

When pure argon was used as the carrier gas, both partial and complete oxidation of methane were observed after pulsing methane to the OC bed at 900 °C and 1 atm (Figure 6). In the seven consecutive pulses with an interval of 10 min, both the conversion of methane and the amount of CO_2_ produced dramatically decrease in the first four pulses and then gradually approach constants in the last three pulses. On the other hand, the amount of hydrogen produced follows an opposite trend. Specifically, methane conversions are 28%, 13%, and 12% in the first, fourth, and seventh pulses, respectively. The amount of CO_2_ produced in the first pulse is 3.5 times as much as that in the fourth pulse, and the amount of hydrogen produced in the fourth pulse is 6 times as much as that in the first pulse; the former decreases by 1.2 times and latter increases by 1.1 times in the last three pulses.

As Figure 6d shows, the selectivity of CO_2_ is as high as 71% in the first two pulses and higher than 32% in the following five pulses. According to the above discussion, the partial oxidation of methane is favorable over methane combustion when it contacts SrFeO_2.5_ at 900 °C. These observations clearly indicate that most oxygen species involved in methane combustion are not the lattice oxygen of bulk SrFeO_2.5_. On the other hand, if only the surface oxygen species account for methane combustion, they will be quickly consumed because these species operate within a time scale of less than 10 ms. Therefore, we surmise that other oxygen species that perform combustion are located in the surface layer.

For the as-prepared SrFeO_3-δ_, the amount of available lattice oxygen species that perform partial oxidation at 900 °C is 7.93 mmol/g_OC_; thus, the total amount of this type of lattice oxygen in the fixed-bed reactor is about 2.75 mmol. For each pulse, the amount of methane injected into the reactor is around 0.0557 mmol. Because the maximum methane conversion is 28%, it is reasonable to assume that the structure of SrFeO_2.5_ remains intact in the pulse run. Between two pulses, the total amount of molecular oxygen fed to the OC is 8.9 × 10^−2^ μmol in 10 min, which is only 2 percent of minimum amount of oxygen species that participate in methane combustion. Thus, the replenishment of consumed oxygen species by gaseous oxygen can be ignored between pulses. The oxygen species that are located in the surface layer can replenish those involved in methane combustion, which predominantly occurs on the surface of SrFeO_2.5_. If the pulse interval increases, there are more oxygen species that perform methane combustion. As expected, more CO_2_ is produced as the interval changes from 10 to 20 min (Figure 6a). A very interesting observation is that the relationship between the amount of CO_2_ produced and the interval in the last four pulses (t_interval_ = 10, 20, 30, 60 min) is linear (inset in Figure 6c), which may imply that not the diffusion but other factors such as the activation of adsorbed molecular oxygen control the evolution of oxygen species. More studies are required to clarify the evolution mechanism in the future. 

**Figure 7:**
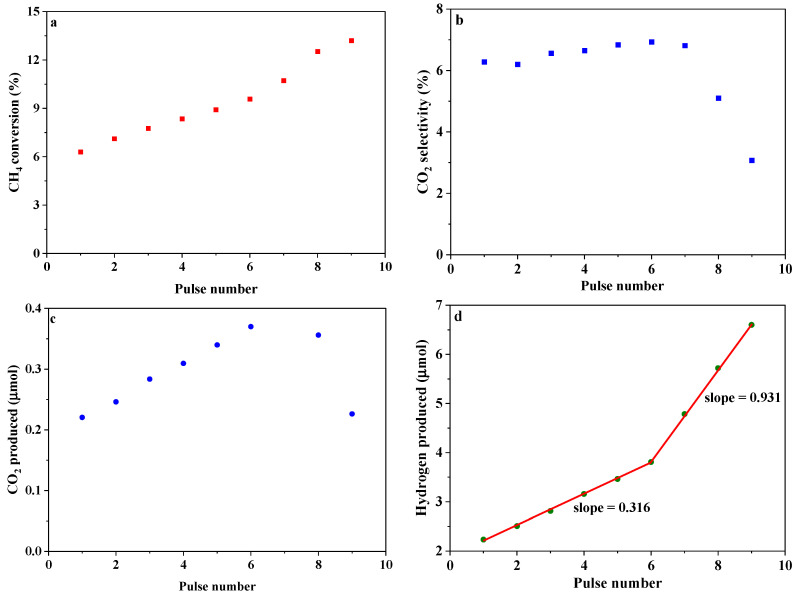
Methane oxidation by SrFeO_2.5_ at 900 °C and 1 atm in a pulse mode: m_OC_ = 0.35 g, F_H2_ = 1 STP mL/min, F_Ar_ = 100 STP mL/min, n_CH4,injected_ = 55.7 μmol, pulse interval = 10 min. (**a**) CH_4_ conversion (%), (**b**) CO_2_ selectivity (%), (**c**) CO_2_ produced (μmol), (**d**) Hydrogen produced (μmol).

When introducing hydrogen into the carrier gas, nine consecutive pulses with an interval of 10 min were carried out, and the results are illustrated in Figure 7. As can be seen, both the conversion of methane and the amount of hydrogen produced monotonically increase with pulse number. On the other hand, the amount of CO_2_ produced increases in the first seven pulses and then decreases in the last three pulses. A close examination of Figure 7d reveals the variation of the amount of hydrogen produced with pulse number is linear and includes two segments; the slope of the second segment (6–9 pulse) is almost 3 times that of the first segment (1–6 pulse). At this moment, both where this linear relationship comes from and what causes the change in the slope remain elusive. The amount of CO_2_ produced with hydrogen in the carrier gas is much less than that without hydrogen in the carrier gas, decreasing by more than one order of magnitude. Specifically, a decrease by a factor of 50 was observed in the first pulse. Assuming that only methane oxidation occurs after injecting it into the OC bed, then the average mole ratio of CO produced to CO_2_ generated in the first 8 pulses falls between 14 and 19, but it becomes 32 in the ninth pulse. The latter is very close to the one (37.4) predicted by the thermodynamic analysis. These observations unambiguously confirm the above assumption that some oxygen species that perform combustion are located in the surface layer. Furthermore, the combustion of methane by these oxygen species may follow the bulk nucleation-growth model, which is frequently used to describe methane partial oxidization by bulk lattice oxygen [25,26,27].

## 4. Conclusions

The perovskite (SrFeO_2.86_) prepared by the Pechini-type polymerizable complex method has a tetragonal structure and may contain a minor amount of orthorhombic phase at ambient conditions. When heating SrFeO_2.86_ from 100 to 900 °C in pure argon that contains roughly 2 ppmv molecular oxygen, it first turns into SrFeO_2.75_ and subsequently into SrFeO_2.5_; the activation energy of the first step is 1.7 times as high as that of the second one. 

When methane was brought into contact with SrFeO_2.5_ at 900 °C and 1 atm in a pulse mode, the presence of hydrogen in the carrier gas significantly suppressed methane combustion. However, it cannot totally eliminate oxygen species that perform complete oxidation. In the absence of hydrogen, the oxygen species involved in methane combustion can be partially replenished by extending the pulse interval. The restored species predominantly originate from those that reside in the surface layer or even in the bulk. The replenishment is controlled by other factors other than diffusion; the detailed mechanism could be revealed by combining more experimental investigations and theoretical studies.

## Data Availability

Data will be made available on request.
